# Long overdue: a new, widespread, species of *Pristimantis* (Anura, Strabomantidae) from the Chocó region and the synonymy of *Pristimantis subsigillatus*

**DOI:** 10.3897/zookeys.1286.194383

**Published:** 2026-07-24

**Authors:** Santiago R. Ron, Poulette Paredes-Aguirre, Diego A. Paucar, Yaara López, Katherine Apunte, Jhael A. Ortega

**Affiliations:** 1 Museo de Zoología, Centro de Investigaciones de la Biodiversidad CIBIO, Facultad de Ciencias Exactas, Naturales y Ambientales, Pontificia Universidad Católica del Ecuador, Av. 12 de Octubre y Roca, Aptdo. 17-01-2184, Quito, Ecuador Museo de Zoología, Centro de Investigaciones de la Biodiversidad CIBIO, Facultad de Ciencias Exactas, Naturales y Ambientales, Pontificia Universidad Católica del Ecuador Quito Ecuador; 2 Current address: Department of Biological Sciences, Virginia Tech, Blacksburg, Virginia, USA Department of Biological Sciences, Virginia Tech Blacksburg United States of America

**Keywords:** Andes, holotype, integrative taxonomy, lectotype, phylogeny, systematics

## Abstract

As currently defined, *Pristimantis subsigillatus* is a species of the *P. lacrimosus* species group distributed in the Chocó of Colombia and Ecuador, from lowlands to Andean foothill forests. Upon re-examination of the holotype of *P. subsigillatus*, we conclude that the populations currently ascribed to *P. subsigillatus* are not conspecific with the holotype. Instead, the holotype is conspecific with one of the syntypes of *P. latidiscus*, a species described four years before from a nearby locality. To avoid the ambiguity in the application of the binomen *Hylodes latidiscus*, we designate a lectotype. As a result, *P. subsigillatus* becomes a junior synonym of *P. latidiscus*. The misidentified populations represent an undescribed species which we describe based on morphological, bioacoustic, and genetic data as *Pristimantis milpe***sp. nov**. The new species has one of the widest distributions among Chocoan *Pristimantis* and is unusual for having sexual dimorphism in coloration. It is most closely related to *Pristimantis degener* from which it diverged in the late Miocene and is morphologically distinct. The fact that such a common species remained “invisible” to taxonomy highlights the importance of correctly establishing the link between the name-bearing types and natural populations. Finally, we discuss how taxonomic treatments of *Pristimantis* during the second half of the 20^th^ century often made this non-trivial error.

## Introduction

*Pristimantis* is the most speciose vertebrate genus and is composed of 635 species distributed from Central America to Bolivia, northern Argentina, and Brazil (Frost 2026). The countries with the highest diversity of *Pristimantis* are Ecuador (274 species) and Colombia (224 species) (Frost 2026). Most species inhabit the Andean mid and high elevations, but some groups have also diversified in the lowlands of the Amazon and Chocó regions. One of them is the *Pristimantis lacrimosus* species group (Ron et al. 2020; Carrión-Olmedo and Ron 2021).

The *P. lacrimosus* species group has undergone several taxonomic treatments in the past ten years (Arteaga et al. 2013; Rivera-Correa and Daza 2016; Gonzalez-Duran et al. 2017; Ron et al. 2020; Carrión-Olmedo and Ron 2021). The available evidence suggests that the group originated in the Chocó region (Ron et al. 2020) and, as currently defined, has 43 described species (Castillo-Urbina et al. 2023; Mônico et al. 2025). Among them, *P. subsigillatus* (Boulenger 1902) (described as *Hylodes subsigillatus*) has a wide geographic range in the Chocó of Ecuador and Colombia (Arteaga et al. 2016). The holotype is an adult female from Salidero, Esmeraldas Province, Ecuador. After its description, *P. subsigillatus* was absent in the literature until Lynch (1980b) redescribed it based mainly on specimens collected in Ecuador (Esmeraldas, Pichincha, and Santo Domingo de los Tsáchilas provinces). All subsequent treatments of the species followed Lynch (1980b) characterization. Lynch and Duellman (1997) presented a species account and Hedges et al. (2008) transferred it to its current genus. *Pristimantis subsigillatus* was subject of a phylogeographic analysis showing a large distribution range, relative to other *Pristimantis*, and low genetic variation among populations (Arteaga et al. 2016). Neither Lynch (1980b), Lynch and Duellman (1997), nor Arteaga et al. (2016) suggested taxonomic problems within the species. While we were working on this revision, Coloma and Duellman (2025) suggested that *P. subsigillatus* could be a species distinct from the one currently assigned to that name. However, they did not provide molecular evidence nor noted the conspecificity between the type material of *P. subsigillatus* and *P. latidiscus* (Boulenger, 1898) (see Results).

In this study, we present a taxonomic review of *Pristimantis subsigillatus* based on a reexamination of its type material and that of *P. latidiscus*. We conclude that Lynch (1980b) redescription pertain to a species distinct from the holotype. Therefore, most populations currently assigned to *P. subsigillatus* have been incorrectly identified and actually belong to an unnamed species. Based on morphological, bioacoustic, and genetic data, we describe the new species. We also designate a lectotype for *Hylodes latidiscus* (= *Pristimantis latidiscus*) which renders *Hylodes subsigillatus* its junior synonym.

## Materials and methods

### Morphological comparisons and descriptions

Only adult specimens were used for the comparisons. We examined specimens of the new species deposited in the collections at Museo de Zoología (**QCAZ**) of Pontificia Universidad Católica del Ecuador and morphologically similar species from the Ecuadorian Chocó. Our comparisons also include the type specimens of *Pristimantis subsigillatus* and *P. latidiscus* deposited at the Natural History Museum of London (**NHMUK**). Examined material is listed in Suppl. material 1. Specimens deposited at the QCAZ collection were fixed in 10% formalin and are stored in 70% ethanol.

Sex was determined through gonadal inspection, or by the presence of vocal slits and vocal sacs. Adulthood in males, was assessed by the presence of vocal sacs and vocal slits. Adulthood in females was determined by the presence of convolutions of the oviducts or the presence of large ovarian eggs, following Lynch and Duellman (1997).

Morphological terminology follows Duellman and Lehr (2009). For the Definition and Diagnosis sections, we complement verbal descriptions of coloration with HTML hex codes (in brackets) which can be easily checked online (e.g., https://g.co/kgs/cXHNGh9). To characterize the species color variation, both in life and in preservative, we prefer to provide high-resolution photographs of several individuals instead of verbal descriptions. To unequivocally document the identity of the new species, we accompany our morphological descriptions of the holotype with its DNA sequences.

The following morphological variables were measured in the type material: **SVL** (snout-vent length), **TL** (tibia length), **FL** (foot length, distance from the proximal margin of the inner metatarsal tubercle to the tip of toe IV), **HL** (head length, distance from the angle of the jaw to the snout tip), **HW** (head width at the level of the jaw articulation), **END** (eye-nostril distance, distance between the anterior border of the orbit and the posterior margin of the narial opening), **TD** (tympanum diameter, longest antero-posterior distance between the peripheral edges of the tympanic ring), **IND** (internarial distance, distance between the inner edges of the narial openings), and **IOD** (interorbital distance, breadth of the brain-case between the orbits). Measurements were made using digital calipers (± 0.01 mm).

Conditions for the relative length of toes III and IV follows Lynch and Duellman (1997). Condition of the tympanum are as follows: (A) tympanic membrane and tympanic annulus prominent; (B) tympanic membrane not differentiated and tympanic annulus visible along more than 50% of its perimeter; (C) tympanic membrane not differentiated and tympanic annulus only visible ventrally and, (D) tympanic membrane not differentiated and tympanic annulus absent (modified from Lynch and Duellman 1997). To examine the discoidal fold, a ventral incision was made in the pelvic region following the methodology of Taboada et al. (2013).

### Phylogenetic analyses and genetic distances

For the molecular phylogenetic analyses, we obtained DNA sequences for the mitochondrial genes 12S and 16S RNA, tRNA^Leu^, NADH dehydrogenase subunit 1 (ND1), tRNA^Ile^, tRNA^Gln^, and tRNA^Met^. We also included the nuclear gene Recombinase-Activating Gene (RAG1). Tissues were obtained from the genome bank of the Museum of Zoology of the Pontifical Catholic University of Ecuador (QCAZ). DNA was extracted from liver or muscle tissue preserved in 95% ethanol using a modified guanidine thiocyanate protocol (M. Fujita, unpublished). PCR reactions were performed using 16L19 and 16H36E primers for 16S (Heinicke et al. 2007), 12Sh and 12SKH for 12S (Goebel et al. 1999), 16S-frog and t-Met-frog for ND1 and tRNAs (Wiens et al. 2005), and R182 and R270 (Heinicke et al. 2007). Amplicons were sequenced by Macrogen (Macrogen Inc., Seoul, Korea). Sequences were assembled in GeneiousPro 9.1.8 (Kearse et al. 2012). Vouchers and GenBank accession numbers for newly generated sequences are shown in Table 1.

**Table 1.** Newly generated DNA sequences used in phylogenetic analyses.

**Species**	**Voucher**	**RAG1**	**ND1**	**16S**	**12S**
*P. degener*	QCAZ-A 65567	PZ282023		PZ229109	
*P. degener*	QCAZ-A 66324	PZ282024		PZ229110	
*P. eremitus*	QCAZ-A 43390	PZ282026		PZ229111	
*P. milpe* sp. nov.	QCAZ-A 49366			PZ229112	
*P. milpe* sp. nov.	QCAZ-A 55069	PZ282027		PZ229113	
*P. milpe* sp. nov.	QCAZ-A 65562	PZ282028		PZ229114	
*P. milpe* sp. nov.	QCAZ-A 67342	PZ282029		PZ229115	
*P. milpe* sp. nov.	QCAZ-A 67424	PZ282030		PZ229116	
*P. milpe* sp. nov.	QCAZ-A 74169	PZ282031		PZ229117	
*P. milpe* sp. nov.	QCAZ-A 77106	PZ282032	PZ282022	PZ229118	PZ229134
*P. nyctophylax*	QCAZ-A 15310	PZ282033		PZ229119	
*P. nyctophylax*	QCAZ-A 31319	PZ282034		PZ229120	
*P. nyctophylax*	QCAZ-A 35359	PZ282035		PZ229121	
*P. nyctophylax*	QCAZ-A 36739	PZ282036		PZ229122	
*P. nyctophylax*	QCAZ-A 36741	PZ282037		PZ229123	
*P. nyctophylax*	QCAZ-A 48091	PZ282038		PZ229124	
*P. nyctophylax*	QCAZ-A 49638	PZ282039		PZ229125	
*P. nyctophylax*	QCAZ-A 62898	PZ282040		PZ229126	
*P. nyctophylax*	QCAZ-A 67675	PZ282041		PZ229127	
*P. nyctophylax*	QCAZ-A 70560	PZ282042		PZ229128	
*P. nyctophylax*	QCAZ-A 72334	PZ282043		PZ229129	
*P. eremitus*	QCAZ-A 24827	PZ282044		PZ229130	
*P. latidiscus*	QCAZ-A 55044	PZ282045		PZ229131	
*P. latidiscus*	QCAZ-A 55066	PZ282046		PZ229132	
*P. eremitus*	QCAZ-A 40002	PZ282025	PZ282021		

We complemented our newly generated sequences with sequences from GenBank (Lynch and Duellman 1997; Heinicke et al. 2007; Padial et al. 2007; Hedges et al. 2008; Garcia et al. 2012; Pinto-Sanchez et al. 2012; Arteaga et al. 2013, 2016; Rivera-Prieto et al. 2014; Hutter and Guayasamin 2015; Ortega-Andrade et al. 2015; Chavez and Catenazzi 2016; Rivera-Correa and Daza 2016, 2020; Shepack et al. 2016; Guayasamin et al. 2017; Paez and Ron 2019; Reyes-Puig et al. 2020; Ron et al. 2020; Sánchez-Nivicela et al. 2020; Carrión-Olmedo and Ron 2021; Bejarano-Muñoz et al. 2022). The phylogenetic analyses were based on a matrix of 375 individuals and 4584 bp of DNA sequences of the mitochondrial genes (including flanking tRNAs) 12S rRNA (1033 bp), 16S rRNA (1752 bp, partial sequence), ND1 (1164 bp), and the nuclear gene RAG1 (632 bp). The alignment of the sequences was performed in GeneiousPro 9.1.8 (Kearse et al. 2012) with the plug-in MAFFT (Katoh and Standley 2013) and a posterior manual alignment with Mesquite v. 3.61 (Maddison and Maddison 2019). The aligned matrix is available in Zenodo under DOI https://doi.org/10.5281/zenodo.19439699 (https://shorturl.at/IvcFs).

Phylogenetic relationships were inferred for all genes concatenated using maximum likelihood (ML) as optimality criterion. We partitioned the matrix by gene and codon position and each partition was analyzed under model GTR + R + I. The phylogenetic search was carried out in software IQ-TREE multicore v. 2.2.0 (Minh et al. 2020) under default settings. To assess branch support, we made 1000 ultrafast bootstrap searches (-bb 1000 command) and 1000 replicates for the SH-like approximate likelihood ratio test (-alrt 1000 command) (Guindon et al. 2010). We considered that branches with bootstrap values > 94 and SH-aLRT values > 79 had strong support.

To estimate a time-tree, we applied the least square dating method (To et al. 2016) using IQ-TREE dating option. We used one calibration point, based on the phylogeny of Hime et al. (2020): 47.1 My for the divergence between Eleutherodactylidae (the outgroup in our analysis) and *Pristimantis*.

To calculate uncorrected p-distances for the gene 16S rRNA, we used the package Ape 5.0 (Paradis and Schliep 2019) in R version 4.5.2 (R Core Team 2025). We only report distances for sequences overlapping by more than 500 bp. We applied pairwise deletion in all comparisons.

### Bioacoustic analyses

Advertisement calls recordings were made with a Sennheiser™ ME-67 directional microphone with digital recorder Olympus™ LS10. The only exception was one individual recorded with a Huawei P20 Pro phone (audio extracted from video). Calls were analyzed using software Raven 1.6 (Center for Conservation Bioacoustics 2019) at a sampling rate of 48.0 kHz and a frequency resolution of 11.7 Hz. The time grid had 50% of overlap and a hop size of 5.33 ms. If available, several calls were analyzed per individual to obtain an average. Original recordings are deposited in the audio archive of Museo QCAZ and are available through the BIOWEB, Anfibios del Ecuador website (https://bioweb.bio). We applied Köhler et al. (2017) call centered terminology.

### Conservation status

To assess the conservation status of the new species, we applied the Red List IUCN criteria (IUCN Standards and Petitions Subcommittee 2017). We estimated the extent of occurrence based on all known localities using the R-package redlistr v. 1.04.

## Results

### Review of the type specimens of *Hylodes latidiscus*Boulenger 1898 and *Hylodes subsigillatus*Boulenger 1902

**Figure 1.**
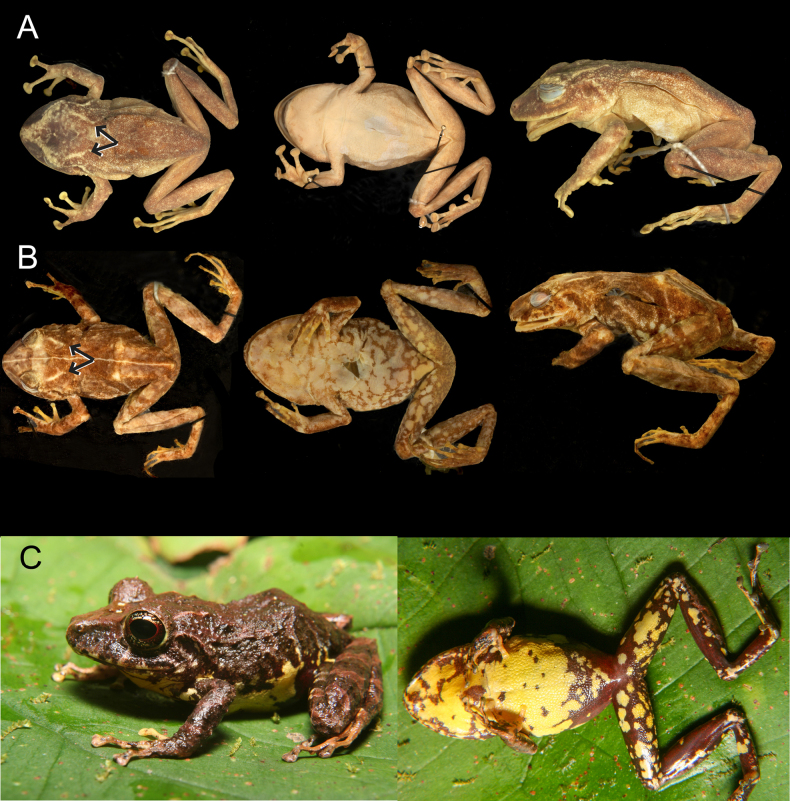
*Pristimantis latidiscus* and *Pristimantis subsigillatus*. **A**. Lectotype (by present designation) of *Pristimantis latidiscus* BMNH 1947.2.15.66; **B**. Holotype of *Pristimantis subsigillatus* BMNH 1947.2.17.1; **C**. Live individual of *P. latidiscus*QCAZ-A 32128 from San Francisco, Durango road, Esmeraldas Province. Photographs of types in dorsal, ventral, and lateral views (from left to right). Note scapular ridges in both holotypes (arrows) and similarity of ventral pattern between the holotype of *P. subsigillatus* and *P. latidiscus*QCAZ-A 32128.

The type material of *Hylodes latidiscus* Boulenger, 1898 (= *P. latidiscus*) consists of two syntypes, BMNH 1947.2.15.66 and BMNH 1947.2.15.67, collected in “Cachabé”, Esmeraldas Province, Ecuador by W. Rosenberg (Boulenger 1898); each syntype represents a different species. Syntype BMNH 1947.2.15.66 (Fig. 1) is an adult female, SVL = 55.02 mm with clear postocular ridges, widely expanded finger discs, shagreen dorsal skin with scattered tubercles, supratympanic fold, and tympanum condition B. Its external morphology falls within the variation of published accounts of *Pristimantis latidiscus* (Lynch and Duellman 1997; Yanez-Muñoz et al. 2026).

**Figure 2.**
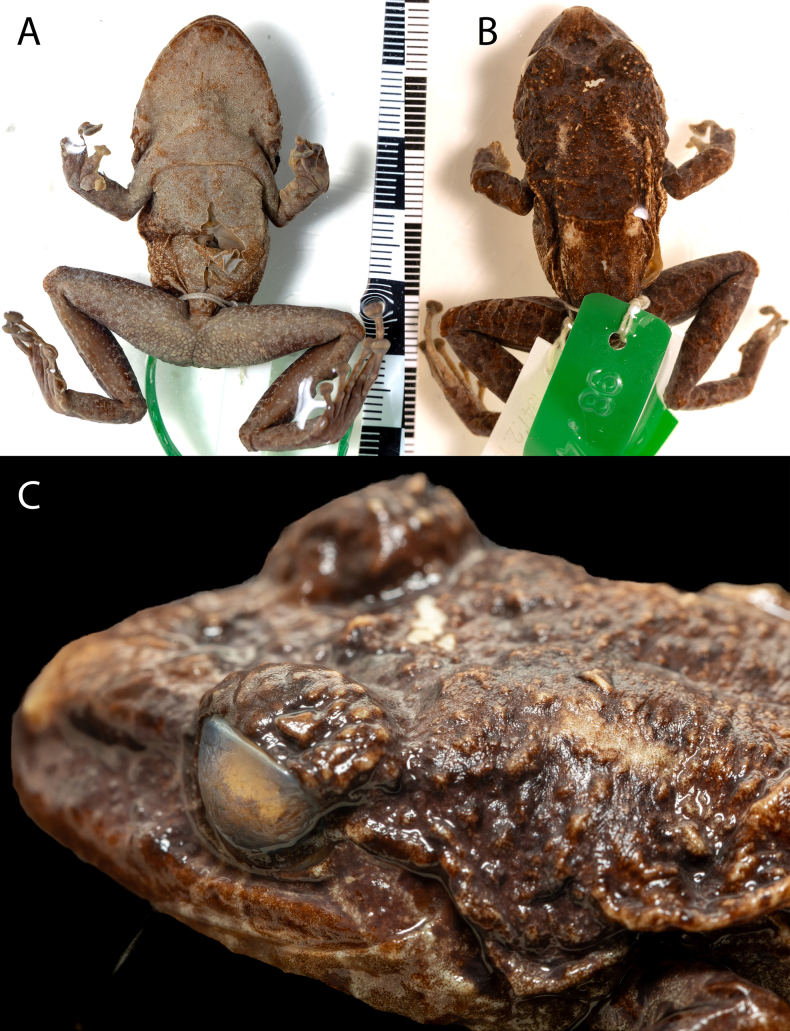
Paralectotype (by present designation) of *Pristimantis latidiscus* BMNH 1947.2.15.67. **A**. Ventral and **B**. Dorsal views of the body; **C**. Dorsolateral view of the head. Note prominent supratympanic fold, small tympanum size, and tuberculated skin. Adult female, SVL = 50.7 mm. Photos by SRR.

The second syntype of *Hylodes latidiscus*, BMNH 1947.2.15.67 (Fig. 2), is an adult female, SVL = 50.7 mm, lacking postocular ridges, with widely expanded finger discs, tuberculate dorsal skin, large tubercles on the eyelids, prominent supratympanic fold, and small and inconspicuous tympanum (20% of eye diameter; tympanum condition C). It represents a distinct species from syntype BMNH 1947.2.15.66 from which it differs by having more tuberculate dorsal skin, large tubercles on the eyelids, and smaller and less conspicuous tympanum. The variation falls outside the known range for *P. latidiscus* (sensu Lynch and Duellman 1997; Yanez-Muñoz et al. 2026). Based on its morphology and collection locality, BMNH 1947.2.15.67 can be confidently assigned to *P. cisnerosi* Reyes-Puig, Yánez-Muñoz, Ortega & Ron, 2020.

Because the syntypes of *Hylodes latidiscus* represent two different species, a lectotype designation is needed to avoid the ambiguity in the application of the species name. Therefore, herein we designate as lectotype of *Hylodes latidiscus* specimen BMNH 1947.2.15.66. Therefore, BMNH 1947.2.15.67 automatically becomes a paralectotype. We chose as lectotype BMNH 1947.2.15.66 instead of BMNH 1947.2.15.67 to favor taxonomic stability. The alternative would require a change to the widely used name “*P. latidiscus*” for “*P. subsigillatus*”, a name incorrectly associated with a different species. In addition, it would invalidate *P. cisnerosi* as it would become a junior synonym of *P. latidiscus*. By choosing as lectotype BMNH 1947.2.15.66, we retain the current use of *P. latidiscus* and *P. cisnerosi* and only invalidate *P. subsigillatus*. Our choice also follows Recommendation 74A of the Code of Zoological Nomenclature: “Agreement with previous restriction. In designating a lectotype, in order to preserve stability of nomenclature an author should act consistently with, and in any event should give great weight to, previously accepted taxonomic restrictions of the application of the name.” In the case at hand, BMNH 1947.2.15.66 corresponds to most species accounts for *P. latidiscus* published during the last 30 years (e.g., Lynch and Duellman 1997; Yanez-Muñoz et al. 2026). As far as we know, there is not a single published identification of *P. latidiscus* that corresponds to the species of paralectotype BMNH 1947.2.15.67 which may explain why it was described as *P. cisnerosi* by Reyes-Puig et al. (2020).

**Figure 3.**
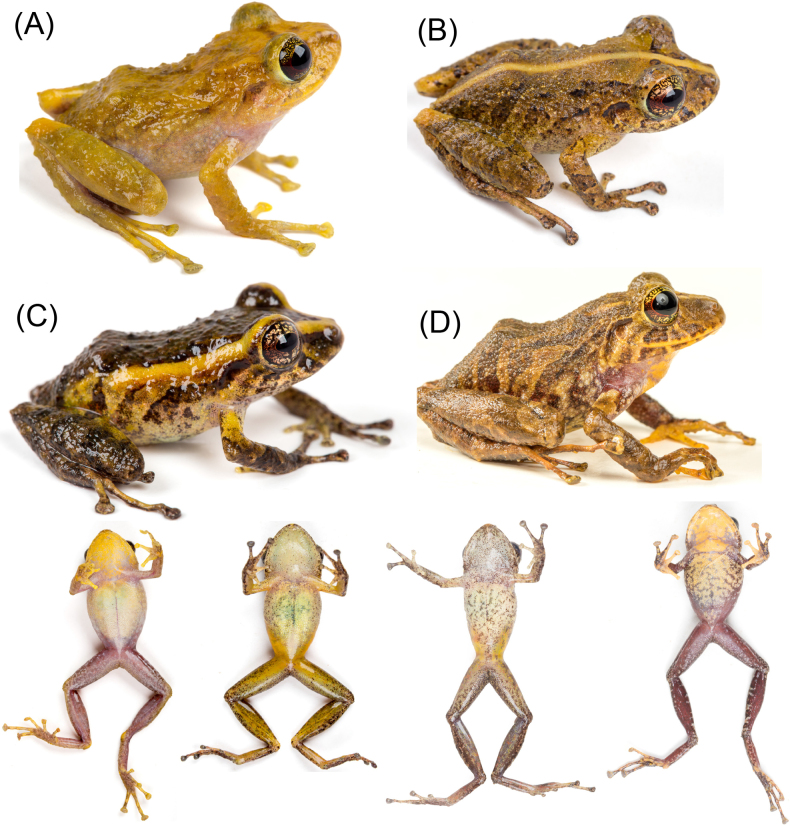
Morphological variation in adult *Pristimantis latidiscus*. First and second rows, dorsolateral views; third row ventral views. **A**. QCAZ-A 66612, adult female, SVL = 34.0 mm; **B**. QCAZ-A 66613, adult male, SVL = 24.3 mm; **C**. QCAZ-A 66618, adult male, SVL = 20.1 mm; **D**. QCAZ-A 65494, adult female, SVL = 39.0 mm. Ventral views are shown in the same order. All from Ecuador, Esmeraldas Province; QCAZ-A 66612–13 and 66618 from Durango; QCAZ-A 65494 from Reserva Tesoro Escondido.

**Figure 4.**
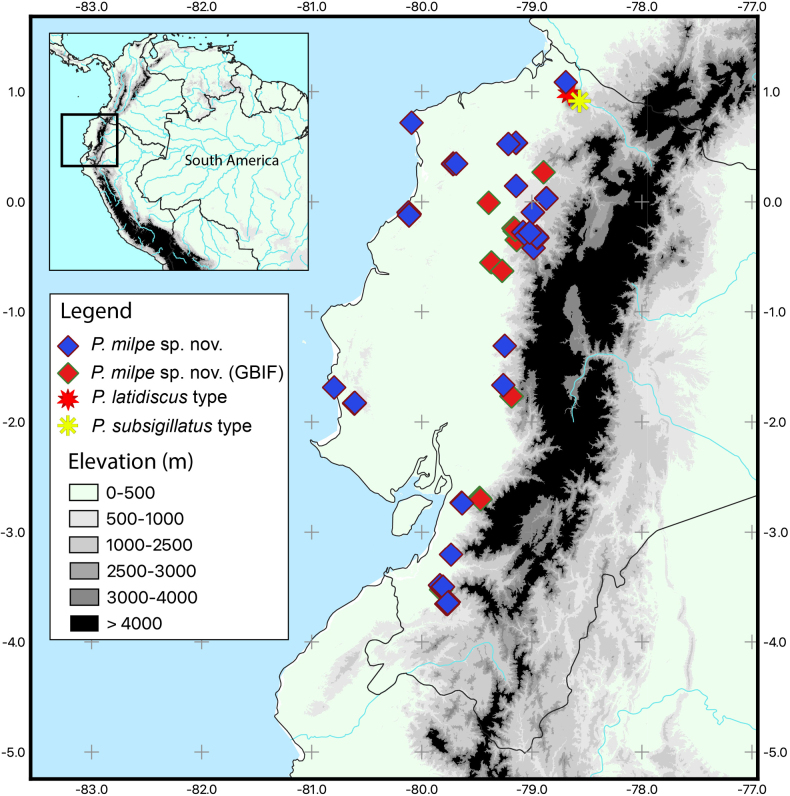
Distribution of *P. milpe* sp. nov. and the type localities of *P. latidiscus* and *P. subsigillatus*. Localities of *P. milpe* sp. nov. are based on museum specimens. Blue diamonds are localities with specimens with confirmed identification (Museo de Zoología Pontificia Universidad Católica del Ecuador, QCAZ); red diamonds represent localities obtained from the GBIF database (identified as “*P. subsigillatus*”) from the Museum of Comparative Zoology Harvard University (MCZ), Museo Universidad Técnica Particular de Loja (MUTPL), Museo de Zoología Universidad San Francisco de Quito (ZSFQ), Museo de Zoología, Universidad Tecnológica Indoamérica (MZUTI), National Museum of Natural History (USNM), and University of Kansas Museum of Natural History (KU). GBIF localities require confirmation. The type localities of *P. latidiscus* and *P. subsigillatus* are also shown with a star (NHMUK).

The holotype of *Hylodes subsigillatus* (= *Pristimantis subsigillatus*), BMNH 1947.2.17.1, is an adult female with oviductal eggs and SVL = 34.0 mm (Fig. 1B). Its type locality is Salidero, Esmeraldas Province, Ecuador. The holotype of *P. subsigillatus* and the lectotype of *P. latidiscus* (by present designation) BMNH 1947.2.15.66 share \ /-shaped postocular folds, slightly areolate dorsal skin, wide discs on fingers, and similar head shape (Fig. 1). *Pristimantis latidiscus* has high morphological variability (Fig. 3), but its variation encompasses individuals with ventral coloration of contrasting dark brown areas on a clear background, similar to the holotype of *P. subsigillatus* (compare Fig. 1B with Fig. 1C). The only species of *Pristimantis* with postocular folds in the region of the type locality of *P. subsigillatus* is *P. latidiscus*. The airline distance between the type localities of both species is only 13 km (Fig. 4). Based on the combined evidence, we confidently conclude that the holotype of *P. subsigillatus* and the lectotype of *P. latidiscus* belong to the same species. By applying the principle of priority, we conclude that *Hylodes subsigillatus* (= *P. subsigillatus*) is a junior synonym of *Hylodes latidiscus* (= *P. latidiscus*) because the latter was described four years previously.

### Status of the populations assigned to *Pristimantis subsigillatus* by Lynch (1980b), Lynch and Duellman (1997), and Coloma and Duellman (2025)

**Figure 5.**
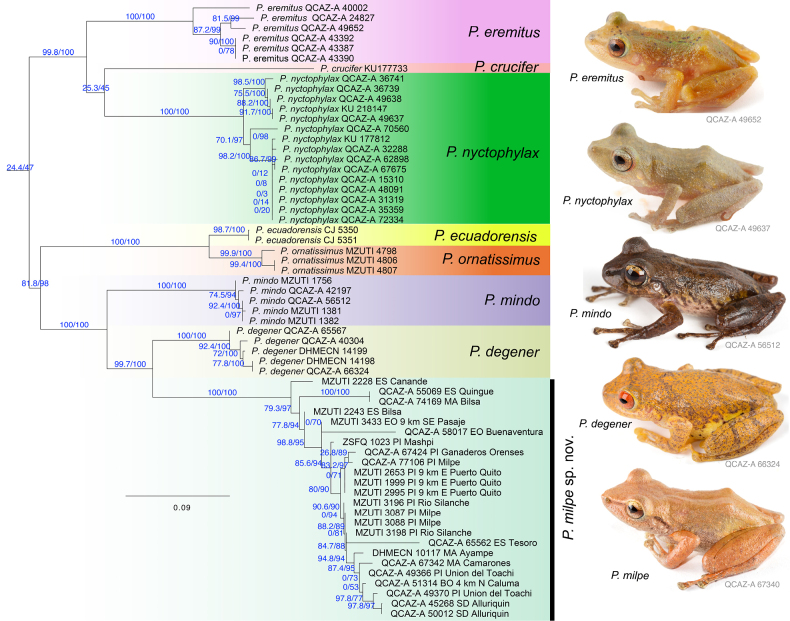
Phylogenetic relationships of the Pacific clade of the *Pristimantis lacrimosus* species group. Maximum likelihood tree obtained for the 16S, 12S, ND1 and RAG1 genes. Branch support (blue numbers) are shown as aLRT values (before the slash) and ultrafast bootstrap values (after the slash). For each individual, the species name is followed by the voucher number. In the new species, the voucher number is followed by the abbreviation of the province and the locality (all in Ecuador). Abbreviations: BO = Bolívar, EO = El Oro, ES = Esmeraldas, MA = Manabí, PI = Pichincha, SD = Santo Domingo. Photographs of the new species and similar species from the Ecuadorian Chocó are shown on the right.

**Figure 6.**
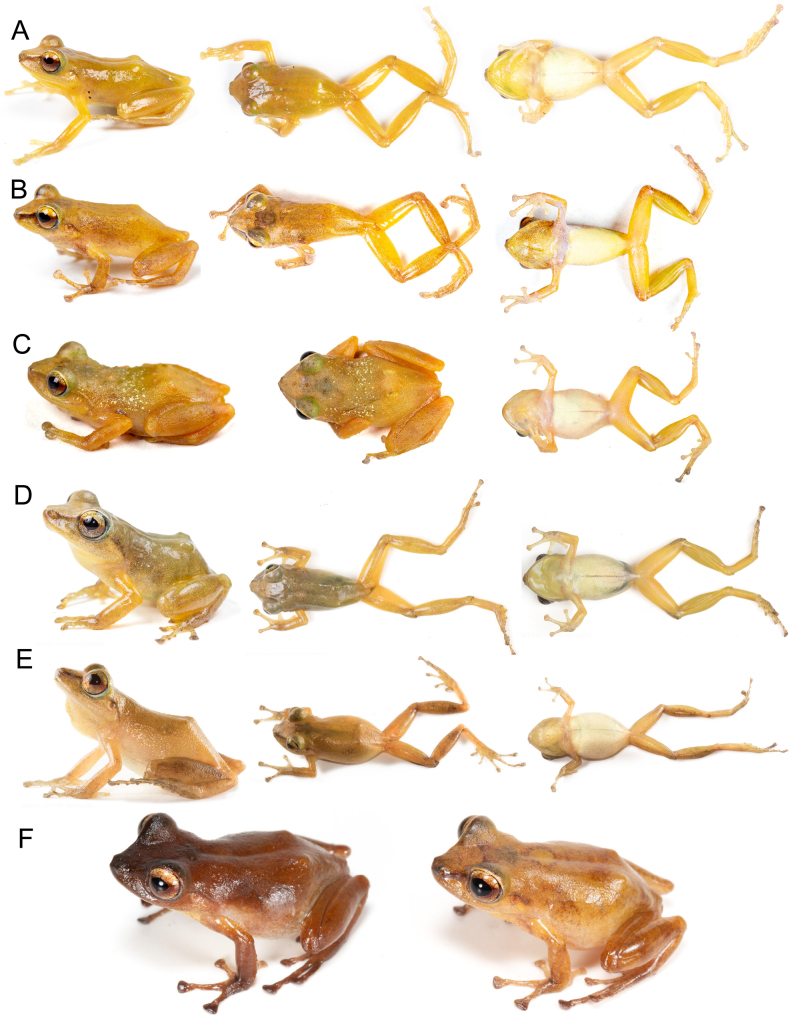
Photographs showing color variation in living adult males of *Pristimantis milpe* sp. nov. **A**. QCAZ-A 77106, holotype, SVL = 24.77 mm; **B**. QCAZ-A 76585, paratype, SVL = 26.97 mm; **C**. QCAZ-A 76518, paratype, SVL = 26.06 mm; **D**. QCAZ-A 65561, SVL = 19.64 mm; **E**. QCAZ-A 67428, SVL = 26.47 mm. Left column: dorsolateral view; central column: dorsal view; right column: ventral view. **F**. QCAZ-A 79899; note significant color change within a period of less than 24 h. Locality data for all specimens is provided in the holotype and paratypes sections.

**Figure 7.**
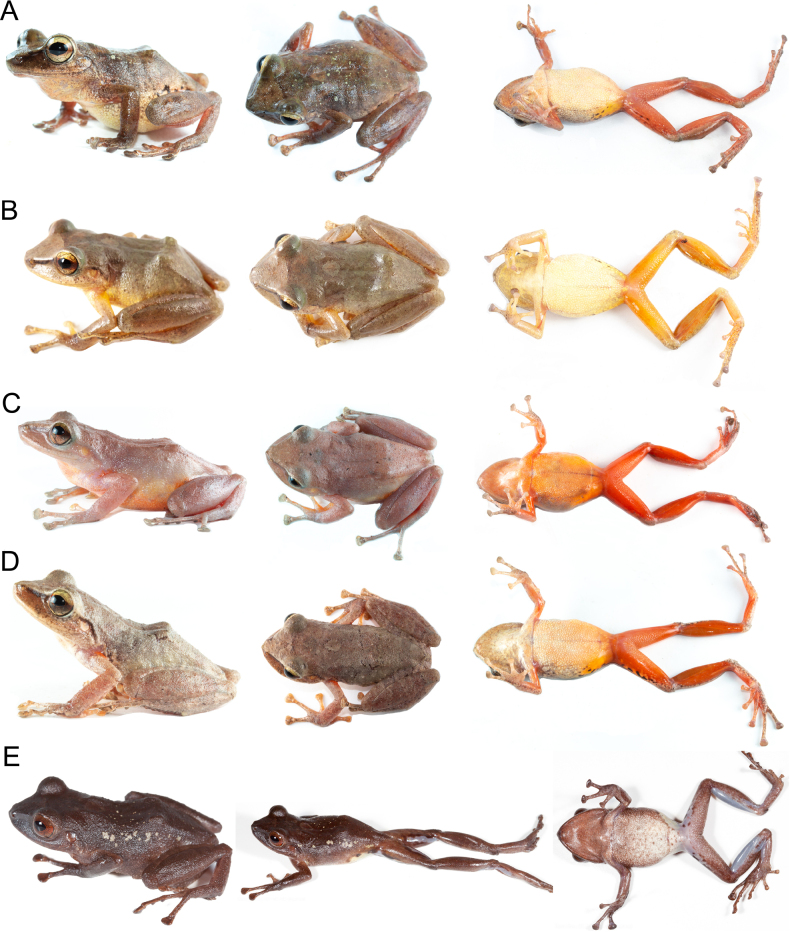
Photographs showing color variation in living adult/subadult females of *Pristimantis milpe* sp. nov. **A**. QCAZ-A 67424, paratype, adult female, SVL = 32.07 mm; **B**. QCAZ-A 45268, paratype, subadult female, SVL = 29.44 mm; **C**. QCAZ-A 74171, paratype, adult female, SVL = 34.21 mm; **D**. QCAZ-A 74172, paratype, adult female, SVL = 34.17 mm; **E**. QCAZ-A 51314, adult female, SVL = 33.13 mm. Left column: dorsolateral view; central column: dorsal view; right column: ventral view. Locality data for all specimens is provided in the paratypes section.

Most populations assigned to “*P. subsigillatus*” by Lynch (1980b) and Lynch and Duellman (1997) are not conspecific with the holotype of *P. subsigillatus* (BMNH 1947.2.17.1; Fig. 1). These populations differ from the holotype in the following (holotype condition in parentheses): (1) absence of postocular folds (\ /-shaped postocular folds present), (2) head protruding in profile (head rounded in profile), (3) Toe V much longer than Toe III; Toe V reaches the distal subarticular tubercle of Toe IV (Toe V slightly longer than Toe III; Toe V does not reach the distal subarticular tubercle of Toe IV), (4) in preservative, venter and ventral areas of limbs white to cream (cream overlain with a brownish-tan, mottled pattern). Based on this evidence, we conclude that the populations assigned to “*P. subsigillatus*” by Lynch (1980b) were misidentified. According to our phylogeny (Fig. 5), they belong to the *P. lacrimosus* species group. Lynch’s misidentification is puzzling given that both species are morphologically very distinct (compare Figs 1, 3 with Figs 6, 7). Lynch’s (1980b) characterization of the species was widely adopted in the subsequent literature (Lynch and Duellman 1997; Hedges et al. 2008; Arteaga-Navarro et al. 2013; MECN-Jocotoco-Ecominga 2013; Arteaga et al. 2016; Rivera-Correa and Daza 2016; Carrión-Olmedo and Ron 2021).

Within the *P. lacrimosus* species group, there are not available binomials (including junior synonyms) that could be applied to those populations. Therefore, they represent an undescribed species that we describe in the following section.

Coloma and Duellman (2025) also noted Lynch’s (1980b) mischaracterization of *P. subsigillatus*. However, they did not realize the conspecificity of the holotype of *P. subsigillatus* with the type material of *P. latidiscus*. Moreover, their species account of “*P. subsigillatus*” is a mixture of at least two species: *P. latidiscus* (e.g., QCAZ-A 27757) and its close relative, *P. rosadoi* (Flores, 1988) (e.g., QCAZ-A 27756; identification corroborated with 16S DNA sequences).

### Phylogenetic relationships

The phylogenetic tree (Fig. 5; Suppl. material 2) is consistent with previous phylogenies for the *P. lacrimosus* species group except for weakly supported nodes (e.g., Ron et al. 2020; Carrión-Olmedo and Ron 2021; Castillo-Urbina et al. 2023). One weakly supported node combines all species from the Pacific basin of Ecuador (same as in the RAxML tree in Portik et al. 2023). In contrast, Ron et al. (2020) and Carrión-Olmedo and Ron (2021) showed them separated in two clades that diverge basally within the *P. lacrimosus* species group. In all phylogenies, however, each clade has strong support: one includes *P. crucifer* (Boulenger, 1899), *P. eremitus* (Lynch, 1980a), and *P. nyctophylax* (Lynch, 1976); the other *P. degener* (Lynch & Duellman, 1997), *P. ecuadorensis* Guayasamin, Hutter, Tapia, Culebras, Peñafiel, Pyron, Morochz, Funk & Arteaga-Navarro, 2017, *P. mindo* Arteaga-Navarro, Yáñez-Muñoz & Guayasamin, 2013, *P. ornatissimus* (Despax, 1911), and the new species (previously reported as “*P. subsigillatus*”).

**Figure 8.**
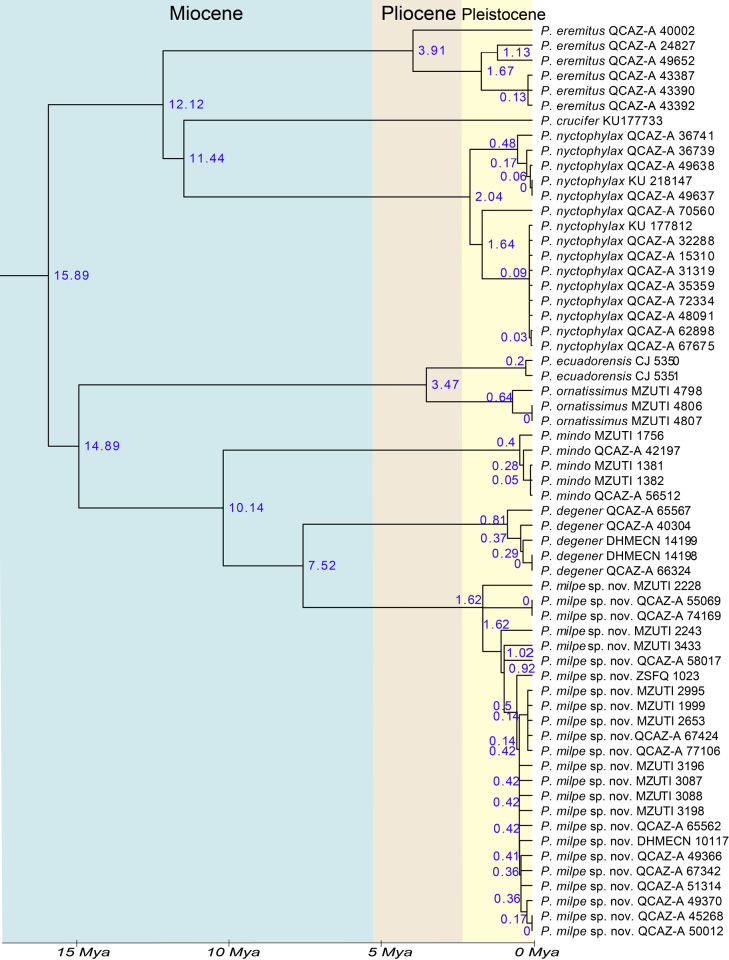
Time-tree for the Pacific clade of the *Pristimantis lacrimosus* species group. The tree is based on sequences of 16S, 12S, ND1, and RAG1 genes. Divergence times, in millions of years (blue numbers) are shown on each node. For each individual, the species name is followed by the voucher number.

According to our time-tree (Fig. 8; Suppl. material 3), most species of the *P. lacrimosus* species group from the Pacific basin originated during the Miocene with the only exception of *P. ecuadorensis* and *P. ornatissimus* which originated in the Pliocene. The new species is more closely related to *P. degener* and *P. mindo*, with strong support. Both species diverged ~7.5 Mya. Sampled individuals are distributed in foothill and lowland Chocoan forests of western Ecuador. The three northernmost populations, Canandé, Quingue, and Bilsa, diverge basally, followed by samples from southern Ecuador (El Oro province). According to our time-tree (Fig. 8), the oldest divergence within the species took place ~1.6 Mya.

**Figure 9.**
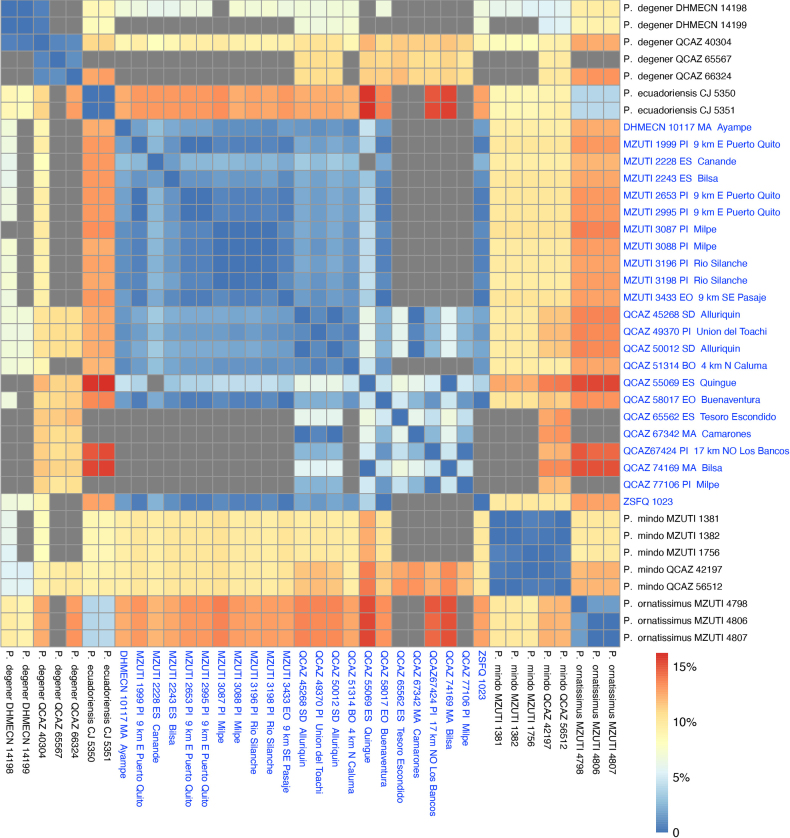
Heatmap of uncorrected p genetic distances for the gene 16S rRNA. Individuals of the new species are shown with blue font. Gray cells indicate comparisons with less than 500 bp.

Genetic distances for the gene 16S are shown in Fig. 9. The average distance between the new species and *P. degener* is 9.2% (range 6.0–12.0%) and with *P. mindo* is 10.5% (range 9.4–14.0%). Intraspecific distances for *P. milpe* sp. nov. are on average 1.8% (range 0–6.6%). The highest distance was observed between Tesoro Escondido (QCAZ-A 65562) and Bilsa (QCAZ-A 74169). This high distance for intraspecific comparisons may be an artifact of sequence quality and the relatively short length of the overlapping fragment (589 bp).

### Systematic account

#### Pristimantis milpe sp. nov.

Taxon classificationAnimaliaAnuraCraugastoridae

B0061B8E-72A9-5208-849D-496C52D445B9

https://zoobank.org/68887E18-6D6A-40EE-9EFD-AA8D23B2D5A8

Figs 5, 6, 7, 11, 12

Eleutherodactylus subsigillatus Lynch, 1980b in part; Lynch and Duellman (1997) in part.
Pristimantis subsigillatus : Arteaga et al. (2013); Arteaga et al. (2016); Chavez and Catenazzi (2016); Rivera-Correa and Daza (2016); Stanescu et al. (2017); Carrión-Olmedo and Ron (2021); Moretta-Urdiales et al. (2025); Fuchs (2025).

##### Type material.

***Holotype***: • QCAZ-A 77106 (field series SC-PUCE 64534), adult male collected in Ecuador, Pichincha Province, Milpe Bird Sanctuary, on the trails of the Reserve (0.031318°N, 78.8673°W), 1150 m, collected by Santiago Ron, María José Navarrete, Julio Carrión, Vincent Premel, and Daniel Zumel on February 12, 2020. ***Paratypes* (*n* = 21)**: • **Ecuador**. Bolívar Province: Tablas La Florida: QCAZ-A 51314, adult female (1.66577°S, 79.25843°W) 361 m, collected by Diana Troya, Francy Mora, Estefanía Boada, Jorge Valencia, and Fernando Ayala on June 09, 2011. • Las Naves Canton, 2 km NE Recinto Naves Chico: QCAZ-A 79899, adult male (1.3091°S, 79.2457°W), 992 m, collected by Edwin Carrillo on October 5, 2024. • Esmeraldas Province: Eloy Alfaro Canton, Santo Domingo de Onzole Parish, Tesoro Escondido Reserve: QCAZ-A 65561, adult male (0.53529°N, 79.14291°W) 294 m, QCAZ-A 65562, adult male, (0.53775°N, 79.14420°W) 309 m, collected by Diego Almeida, Kunam Nusirquia, Diego Paucar, Estefany Guerra, and Diego Quirola on October 19, 2016. • Quinindé Canton, Rosa Zárate Parish, Mache-Chindul Ecological Reserve, Bilsa Biological Station: QCAZ-A 74171, adult female (0.34886°N, 79.71386°W) 506 m, and QCAZ-A 74172, adult female (0.34361°S, 79.71598°W) 526 m, collected by Diego Almeida, Darwin Núñez, Gabriela Pazmiño, Andrea Echeverry, and Kelly Granda on August 9, 2018. • Manabí Province: Jama Canton, Jama Parish, Camarones, Jama-Coaque Ecological Reserve: QCAZ-A 67340, adult female (0.12236°S, 80.11539°W) 646 m, QCAZ-A 67341, adult female, (0.109009°S, 80.10940°W) 640 m, QCAZ-A 67342, subadult female (0.119429°S, 80.11534°W) 661 m, collected by Diego Almeida, Kunam Nusirquia, Darwin Núñez, Fernando Ayala, Malki Bustos, Camila Silva, Valeria Chasiluisa, and Katherin Hinojosa from March 25 to March 28, 2017. • Pichincha Province: La Unión del Toachi, trail to the Botanical Garden: QCAZ-A 49370, adult male (0.3258°S, 78.9490°W) 850 m, collected by Néstor Acosta, Jorge Navarro, John Recalde, Juan Carlos Beción, Scran Najera, Cristina Naranjo, Joaquín Alarcón, Fausto Ocaña, and Marco Vallejo on September 4, 2010. • San Miguel de los Bancos Canton, San Miguel de los Bancos Parish, Ganaderos Orenses: QCAZ-A 67424, adult female, (0.0928°S, 78.9909°W) 726 m, and QCAZ-A 67428, adult male (0.0930°S, 78.9896°W) 707 m, collected by Diego Almeida, Kunam Nusirquia, Darwin Núñez, Fernando Ayala, Malki Bustos, Camila Silva, Valeria Chasiluisa, Katherin Hinojosa, and María del Mar Moretta on March 16, 2017. • San Miguel de los Bancos Canton, Mindo Parish, Milpe Bird Sanctuary: QCAZ-A 76518 and QCAZ-A 76519, adult males (0.0322°N, 78.8683°W) 1137 m, collected by Santiago Ron on February 14, 2019; • QCAZ-A 76585, adult male (0.03168°N, 78.8670°W) 1137 m, collected by Santiago Ron, Yerka Sagredo, Karem López, Adriana Manzano, and Pablo Aceñolaza on December 10, 2018; • QCAZ-A 76819–76820, adult males (0.0319°N, 78.8682°W) 1140 m, collected by Fernando Ayala and Estefany Guerra on November 8, 2018. • QCAZ-A 77104–77105, 77107 adult males, (0.0350°N, 78.8671°W) 1078 m, collected by Santiago Ron, María José Navarrete, Julio Carrión, Vincent Premel, and Daniel Zumel on February 12, 2020. • Santo Domingo de los Tsáchilas province: San José de Alluriquín Canton, Tinalandia Hotel trails: QCAZ-A 45268, adult female (0.2987°S, 79.0523°W) 722 m, collected by Steven Poe and Fernando Ayala on August 13, 2009; • Tinalandia Hotel: QCAZ-A 50012, adult male (0.3032°S, 79.0520°W) 752 m, collected by Alejandro Arteaga-Navarro on January 2, 2011.

##### Common name.

English: Milpe Rain Frog. Spanish: Cutín de Milpe.

##### Diagnosis.

We assign the new species to the genus *Pristimantis* based on the phylogeny (Fig. 5). Species of *Pristimantis* characterized by: (1) smooth to slightly tuberculate dorsal skin, areolate belly; discoidal fold evident; dorsolateral folds absent; (2) tympanum condition B; tympanic ring covered dorsally by supratympanic fold, tympanic membrane not differentiated; (3) truncate snout in dorsal view and protruding in lateral view; (4) upper eyelid generally without tubercles (occasionally one tiny tubercle present); (5) cranial crests absent; (6) oblique dentigerous processes of vomers; (7) vocal slits present, nuptial pads absent; (8) finger I shorter than finger II; expanded, truncated discs; (9) fingers lacking lateral fringes; (10) ulnar tubercles absent; (11) heel tubercle absent, outer edge of tarsus lacking tubercles; (12) ovoid inner metatarsal tubercle, small outer metatarsal tubercle, supernumerary plantar tubercles present; (13) toes lacking lateral fringes, interdigital membrane absent, toe V longer than toe III; (14) in preservative, dorsum varying from light to dark brown, often with irregular dark marks; belly white to cream, anterior surfaces of thighs cream; females have a distinctive pattern in the groin, cream or brown color with black spots; (15) SVL in adult females 32.1–36.1 mm (*n* = 9), SVL in adult males 24.2–27.0 mm (*n* = 12; Table 2).

**Table 2.** Descriptive statistics for morphometric variables of adult individuals of *Pristimantis milpe* sp. nov. Measurements are given as mean ± SD with range in parenthesis. ‘*n*’ is the number of individuals measured. Abbreviations: SVL = snout-vent length; TL = tibia length; FL = foot length; HL = head length; HW = head width; IOD = interorbital distance; IND = internarial distance; END = eye-nostril distance; TD = tympanum diameter. All measurements are in mm.

**Sex**	**Holotype**	**Males (*n* = 12)**	**Females (*n* = 9)**
**SVL**	24.77	25.80 ± 0.91 (24.24–26.97)	33.87 ± 1.21 (32.07–36.14)
**TL**	12.55	12.48 ± 0.37 (11.75–13.03)	16.50 ± 0.84 (15.19–18.02)
**FL**	12.26	11.64 ± 0.56 (10.29–12.26)	15.16 ± 0.57 (14.48–16.23)
**HL**	8.69	8.80 ± 0.31 (8.44–9.55)	11.87 ± 0.40 (11.34–12.51)
**HW**	9.47	9.51 ± 0.22 (9.16–9.84)	13.16 ± 0.47 (12.32–13.97)
**IOD**	3.15	3.11 ± 0.14 (2.92–3.30)	4.24 ± 0.44 (3.35–4.79)
**IND**	2.12	2.12 ± 0.09 (1.93–2.26)	2.74 ± 0.17 (2.55–3.13)
**END**	2.60	2.94 ± 0.16 (2.60–3.15)	3.99 ± 0.24 (3.54–4.37)
**TD**	1.29	1.37 ± 0.09 (1.27–1.50)	1.78 ± 0.08 (1.65–1.92)

##### Differential diagnosis.

**Figure 10.**
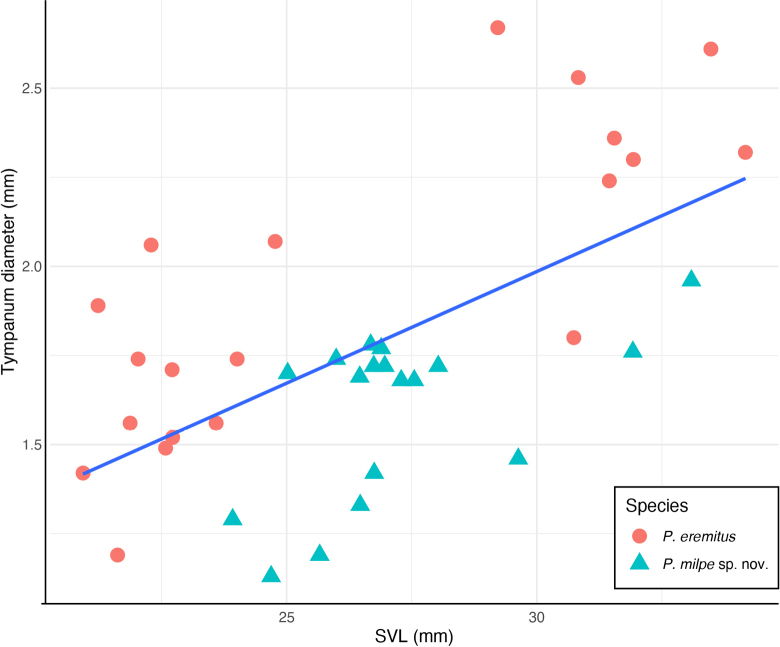
Relationship between tympanum diameter and SVL for adult individuals of *Pristimantis eremitus* and *P. milpe* sp. nov. Note that most *P. eremitus* are above the regression line, indicating a larger tympanum relative to *P. milpe* sp. nov.

In this section, we describe characters for live individuals, unless otherwise noticed. The character states for the new species are described in parenthesis. Among species of the *P. lacrimosus* species group from the Choco region, the most similar is *P. eremitus* which differs by having a predominantly green dorsal coloration (yellow tan, tan olive, to dark brown; Figs 6, 7), more tuberculate dorsal skin, and larger tympanum (Fig. 10). Female *P. eremitus* differ from *P. milpe* by having unpatterned groins (groins orange [edab26] with black spots). Advertisement call differs markedly: *P. eremitus* emits a series of 3–9 notes with an average dominant frequency of 4916 Hz (single note, average dominant frequency 2581 Hz in the new species). *Pristimantis eremitus* call data obtained from Hutter et al. 2016.

The sister species of the new species, *Pristimantis degener* Lynch & Duellman, 1997, differs by having a cream to red iris without contrasting marks (bronze iris [c49969] to dark brown [663333] with a midhorizontal dark brown band [7f4231], with thin black reticulations). The new species is also closely related to *P. mindo*, which differs by having a truncate snout in lateral view (protruding). *Pristimantis nyctophylax* differs from *P. milpe* by having an iris with thick black reticulations and red [e45b2d] to orange [ecbd01] sclera (iris of the new species as described above with light blue [e2e9e2] sclera).

##### Description of the holotype.

Adult male (QCAZ-A 77106); measurements are shown in Table 2. Head as wide as the body, truncate snout in dorsal view and protruding in lateral view; cranial crests absent, tympanic condition B, tympanic annulus covered dorsally by supratympanic fold, tympanic membrane not differentiated. Vocal slits present, near the corner of the mouth, running parallel to the jaw along ~1/3 of its length. Vocal sac folds evident in the gular region; dentigerous processes of vomers present, oblique; nuptial pads absent.

Dorsal skin smooth, dorsolateral folds absent, flank skin without tubercles; throat, chest, and ventral skin areolate; discoidal fold present. Hindlimbs smooth dorsally, thighs finely areolate ventrally.

Low palmar tubercles; well-defined subarticular tubercles, round in ventral and lateral views; all fingers with elongated and slender hyperdistal tubercles; supernumerary tubercles at the base of the fingers; absent lateral fringes. Finger I shorter than Finger II, discs expanded and truncated. Elliptical inner metatarsal tubercle much larger than outer metatarsal tubercle; plantar surface with well-defined supernumerary tubercles, all toes with well-defined hyperdistal tubercles, toes lack lateral fringes; interdigital membranes absent, discs on toes expanded and truncate, toe V much longer than toe III (Toe V reaches the distal subarticular tubercle of toe IV).

In life (Fig. 6A), dorsal coloration is brownish orange [d1990e]; ventral coloration is cream [fdfde5] posterior to the armpits and yellow [fbdd5d] anteriorly. The head has a brown [9c5824] cantal stripe; the iris is bronze [edaf30] with a midhorizontal dark brown [581a04] band. In preservative (Fig. 12A), dorsal coloration is brown [ab8f77]; ventral coloration is light brown [b3aa9b]; the cantal stripe in the head is dark brown [3b2c26].

##### Variation.

**Figure 11.**
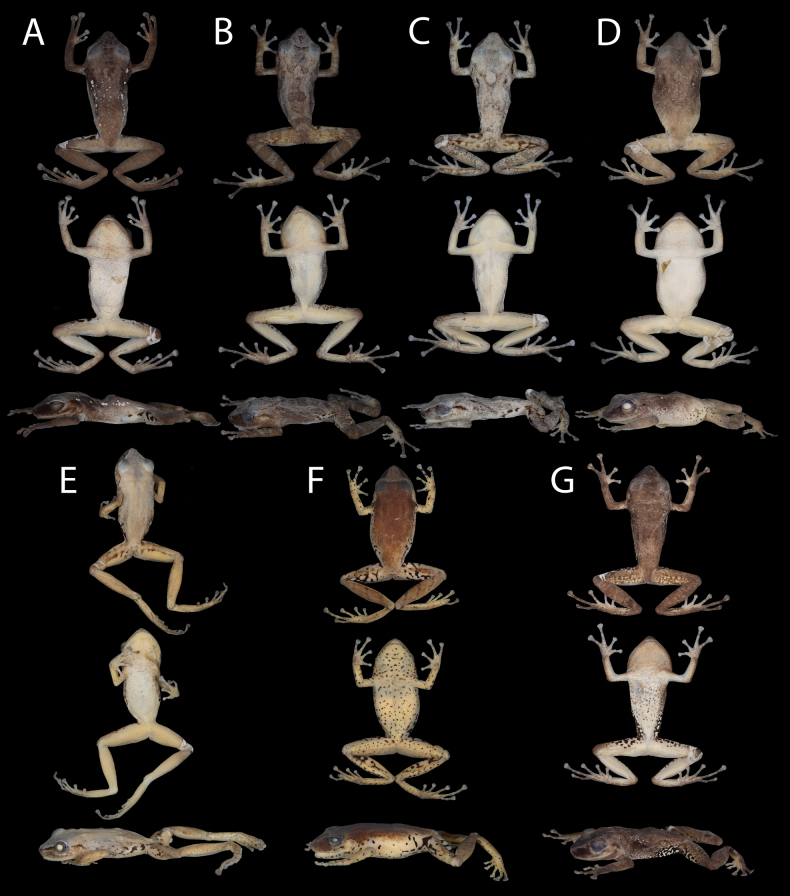
Color variation in preserved adult/subadult females of *Pristimantis milpe* sp. nov. in dorsal, ventral, and lateral views. **A**. QCAZ-A 51314, paratype, adult, SVL = 33.1 mm; **B**. QCAZ-A 55069, subadult, SVL = 27.3 mm; **C**. QCAZ-A 67341, paratype, subadult female, SVL = 27.0 mm; **D**. QCAZ-A 67424, paratype, adult, SVL = 32.1 mm; **E**. QCAZ-A 58018, adult, SVL = 33.1 mm; **F**. QCAZ-A 32919, adult, SVL = 32.9 mm; **G**. QCAZ-A 78832, adult, SVL = 35.4 mm. Photos by Adrián Sunción-Gavilanez. Locality data for all specimens is provided in the paratypes section.

**Figure 12.**
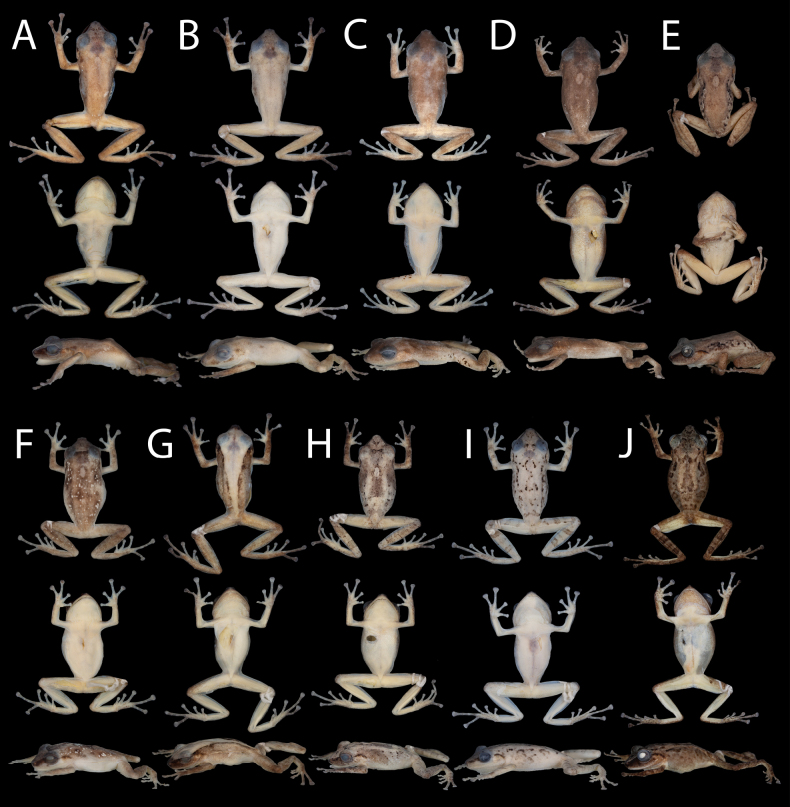
Color variation in preserved adult males of *Pristimantis milpe* sp. nov. in dorsal, ventral, and lateral view. **A**. QCAZ-A 77106, holotype, SVL = 24.8 mm; **B**. QCAZ-A 78622, SVL = 25.6 mm; **C**. QCAZ-A 29791, SVL = 25.3 mm; **D**. QCAZ-A 42346, SVL = 28.0 mm; **E**. QCAZ-A 29319, SVL = 25.9 mm; **F**. QCAZ-A 65562, paratype, SVL = 26.4 mm; **G**. QCAZ-A 77107, paratype, SVL = 25.0 mm; **H**. QCAZ-A 74169, SVL = 22.8 mm; **I**. QCAZ-A 80061, SVL = 28.6 mm; **J**. QCAZ-A 79958. SVL = 26.9 mm. Photos by Adrián Sunción-Gavilanez and Nayely Ramos-Miranda. Locality data for all specimens is provided in the holotype and paratype sections.

Males are smaller than females (adult males average SVL = 25.8 mm, SD = 0.91, range 24.2–27.0 mm, *n* = 12; adult females SVL = 33.9 mm, SD = 1.2, range 32.1–36.1 mm, *n* = 9). Size variation is summarized in Table 2. Details of color variation in life and preservative are shown in Figs 6, 7, 11, 12. There is sexual dimorphism in coloration in life. In females, the groins and hidden surfaces of the thighs are cream, light blue, to orange with black contrasting marks; in males, the groins are unpatterned and have the same coloration as the flanks and ventral areas of the thighs, except for QCAZ-A 29791 which has some dark markings in the groins and anterior surface of the thighs (Fig. 12C), although they are sparser than the markings found in females. Phenotypic plasticity in coloration can be significant; specimen QCAZ-A 79899 varied between dark brown and greenish brown within 24 hours (Fig. 6F). Most specimens have smooth or slightly areolate dorsum with few scattered tubercles, except for QCAZ-A 76518 which has slightly tuberculate skin (Fig. 6C). Although, in preservative, most individuals have a distinct rostral papilla on the tip of the snout, (e.g., QCAZ-A 76518, 76585, 77105), a few individuals lack it (e.g., QCAZ-A 76819–76820, 78832). In the literature, the rostral papilla has been used as a diagnostic character (e.g., Lynch and Duellman 1997) but, given its polymorphism, its utility is limited.

##### Advertisement call.

**Figure 13.**
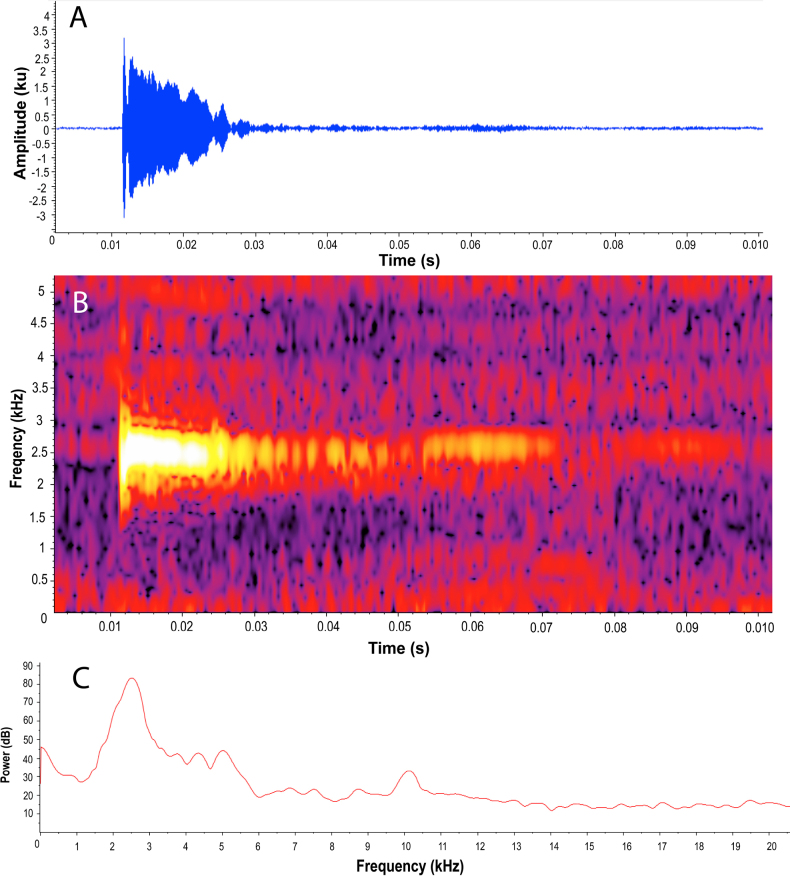
Advertisement call of *Pristimantis milpe* sp. nov. **A**. Oscillogram; **B**. Spectrogram; **C**. Power spectrum. Individual not collected.

The call (Fig. 13; Table 3) is a loud, single tonal click with an average duration of 0.075 s (range 0.062–0.091) and a dominant frequency of 2581.0 Hz (2362.5–2779.3). As is usually the case with dynamic call parameters, the call rate has a wide range of variation, ~3–15 calls per minute. Males call at night over vegetation above the ground (SRR field notes; Lynch and Duellman 1997). Calling males were frequently found on bromeliads or elephant ear plants. The descriptive statistics of our call measurements are similar to those reported by Stanescu et al. (2017) for a population in Provincia de El Oro, Ecuador (reported under “*P. subsigillatus*”). Videos of two calling males at the type locality are available online: https://www.youtube.com/watch?v=w_Rg0dWtTiQ and https://www.youtube.com/watch?v=CKow5VXw4G4.

**Table 3.** Acoustic parameters of calls from five individuals of *Pristimantis milpe* sp. nov. For each specimen, the number of calls analyzed (*n*), mean call duration, peak time, peak time proportion, dominant frequency, and calls per minute are reported. The last row shows descriptive statistics across all individuals: mean, standard deviation (SD), followed by the range in parentheses.

**Specimen**	**Call duration (s)**	**Peak time (s)**	**Peak time proportion**	**Dominant frequency (Hz)**	**Calls per minute**
Not collected (*n* = 4)	0.062	0.0017	0.028	2501.9	6.857
QCAZ 77105 (*n* = 9)	0.072	0.0024	0.034	2630.2	15.499
QCAZ 77104 (*n* = 10)	0.091	0.0170	0.187	2630.9	6.356
QCAZ 76585 (*n* = 6)	0.063	0.0077	0.123	2779.3	2.963
Not collected (*n* = 5)	0.083	0.0028	0.035	2362.5	5.603
Mean, SD, and Range	0.075 ± 0.013 (0.062–0.091)	0.006 ± 0.007 (0.0017–0.0170)	0.081 ± 0.076 (0.028–0.187)	2581.0 ± 113.36 (2362.5–2779.3)	7.456 ± 4.74 (2.96–15.49)

##### Etymology.

The specific epithet is a noun in apposition and refers to the type locality, the Milpe Bird Sanctuary, a private reserve in Pichincha Province, Ecuador. The sanctuary protects ~ 100 ha of Foothill Montane Forest and is part of the Andean Chocó, one of the most diverse and threatened regions on the planet. The Milpe Bird Sanctuary is managed by the Mindo Cloudforest Foundation, a non-profit organization dedicated to the conservation of biodiversity in the Andean Chocó. The Foundation maintains close relationships with local communities, promoting environmental education, sustainable livelihoods, and participatory management of natural resources. It also supports other private reserves and local Decentralized Autonomous Governments in forest restoration through the production of native plants in their nurseries, strengthening long-term conservation in the region.

##### Distribution.

The new species is distributed in the Pacific basin of Ecuador and southern Colombia, from sea level to 1200 m of elevation (Fig. 4). In Ecuador, it occurs in Western Montane Forest, Western Foothill Forest, Choco Tropical Rainforest, and Deciduous Forest (as defined in Ron et al. 2024). It was reported from SW Colombia by Lynch and Duellman (1997), Lynch (1980b), and Lynch and Suárez-Mayorga (2004) as “*Eleutherodactylus subsigillatus*”. Apparently, all reports are based on a single specimen, AMNH 86359, and its identification needs to be confirmed. Its occurrence in Colombia is expected given that localities in northern Ecuador are just 18 km from the border with Colombia. *Pristimantis milpe* has one of the largest distributions of a species of *Pristimantis* in the Chocó region of Ecuador.

##### Natural history.

Most individuals have been found in secondary forests, but they also occur in primary forests, banana plantations, silvopastures, and even urban areas (Lynch 1980b; Fuchs 2025; Pontificia Universidad Católica Del Ecuador 2026). In Canandé, they were most frequent in old growth forest and secondary forest while being absent in pastures and cacao plantations (Fuchs 2025). Activity is nocturnal; frogs perch and call from vegetation. At Canandé, they reproduce year-round, provided that environmental conditions are suitable (Fuchs 2025). At the type locality, an amplectant pair was found on 26 December 2025 at 20:59 h over a leaf 1.5 m above the ground in Terra Firme secondary forest; amplexus is axillary (SRR field notes). A study of amphibians inhabiting epiphytic plants on the forest canopy found three individuals in bromeliads 20 m above the ground (2 adults and 1 juvenile; the adults were reported as “*P. subsigillatus*” and the juvenile as an unidentified *Pristimantis*; Moretta-Urdiales et al. 2025). Males call from bromeliads (e.g., https://www.youtube.com/watch?v=w_Rg0dWtTiQ) or over leaves at heights ranging from 30 cm to more than 10 m. When males are disturbed while calling from bromeliads, they sink in the water (SRR pers. obs.) At Canandé, average call height was 6.6 m, usually higher than other species of *Pristimantis*. They call from dusk to sunrise but calling activity peaks between 20:30 and 22:00 h (Fuchs 2025). *Pristimantis milpe* can be locally abundant (Arteaga-Navarro et al. 2013; Fuchs 2025) but most detections are based on calls heard at heights above 2 m. Juveniles and adults have been found by day in bromeliads, leaflitter, and axils of elephant ear plants (Lynch 1980b; Lynch and Duellman 1997). The available information suggest that reproduction is linked to phytotelmata. Their egg deposition site is unknown, but they presumably are direct developers, as other *Pristimantis*.

##### Conservation status.

Based on the minimum convex polygon, the extent of occurrence for the species is 67,072 km^2^. Given its large extent of occurrence, local abundance, and presence under anthropic habitat disturbance (see above), we recommend assigning this species to the Least Concern Red List category.

## Discussion

With ~67,000 km^2^ of extent of occurrence, *Pristimantis milpe* is one of the most widespread species of *Pristimantis* in the Pacific basin. Moreover, it can be locally abundant and conspicuous due to its loud advertisement call (e.g., Lynch 1980b; Fuchs 2025). Therefore, it is astounding that such a common species remained undescribed until now. The reason for this oversight is one of the most consequential errors in alpha taxonomy: failure to correctly link the name-bearing type with populations of the same species. Other examples of this problem in *Pristimantis* are provided below.

Lynch (1980b) incorrectly considered the holotype of *Hylodes subsigillatus* (= *Pristimantis subsigillatus*; BMNH 1947.2.17.1) as conspecific with populations of *P. milpe*. Although Lynch (1980b) lists BMNH 1947.2.17.1 in the examined material, he overlooked notorious morphological differences with *P. milpe*. Among others, the holotype differs in snout shape, ventral color pattern, and the presence of post-ocular folds (compare Fig. 1B to Figs 11, 12). Conspicuous morphological differences are expected given that they represent species as distantly related as possible within *Pristimantis* (Portik et al. 2023). *Pristimantis subsigillatus* (now a junior synonym of *P. latidiscus*) belongs to the subgenus *Hypodictyon*, the sister group of all other *Pristimantis* and diverged from them (including *P. milpe*) ~24 Mya (Portik et al. 2023). Unfortunately, this type of error in Lynch’s taxonomy of *Pristimantis* was not uncommon. *Pristimantis ockendeni* (Boulenger, 1912), *Pristimantis riveti* (Despax, 1911), and *Pristimantis cajamarcensis* (Barbour & Noble, 1920) provide additional examples.

The case of *P. ockendeni* has been thoroughly reviewed (Elmer et al. 2007; Elmer and Cannatella 2008; Mônico et al. 2022). In summary, the species was described by Boulenger in 1912 based on specimens from SE Peru. It was redescribed by Lynch (1974) with specimens from Amazonian Ecuador (> 1200 km N from the type locality) and the type material. The Ecuadorian populations were, in fact, three morphologically distinct species not closely related to *P. ockendeni* sensu stricto (Elmer and Cannatella 2008; Mônico et al. 2022).

The mischaracterization of populations of *P. riveti*, a species described by Despax in 1911, from specimens in Tungurahua Province, Ecuador, is discussed by Páez and Ron (2019) and provides another example of a redescription of a species based on misidentified populations. Lynch (1979) redescribed *P. riveti* based on populations from southern Ecuador, 400 km south from the type locality. As in the previous example, the holotype is listed in the examined material and represents a different species from the populations in southern Ecuador. Lynch’s (1979) mischaracterization of *P. riveti* was uniformly adopted in the literature for the next 40 years.

Lynch (1969) made the first taxonomic review of *P. cajamarcensis*, a species described by Barbour and Noble (1920) in 1920. Lynch’s redescription has a detailed diagnosis and morphological characterization including morphometric data. The redescription was based on populations from Ecuador, 250 km north from the type locality. A recent phylogeny (Chávez et al. 2025) show that samples of *P. cajamarcensis* collected near the type locality are not closely related to the Ecuadorian populations. Therefore, *P. cajamarcensis* should no longer be considered as occurring in Ecuador.

John Lynch’s contribution to the taxonomy of neotropical amphibians has been massive and is the pillar of current *Pristimantis* systematics. His work includes the descriptions of almost 200 species with thorough morphological accounts frequently based on abundant comparative material. Most species of *Pristimantis* described by Lynch are valid despite the historic limitation of lacking molecular data. However, his work in *Pristimantis* has one non-trivial limitation: often, he paid little attention to the morphology of name-bearing types which are the single objective anchors for the species names. As a result, species described during the 19^th^ and 20^th^ centuries, prior to his work, were often mischaracterized, as exemplified by *P. subsigillatus* and the species discussed above. Moving forward, *Pristimantis* taxonomists are advised to critically examine Lynch’s treatment of old *Pristimantis* binomens by comparing the name-bearing types with the populations currently ascribed to them. These historical oversights serve as a reminder that even common and conspicuous species can remain effectively ‘invisible’ to taxonomy until the fundamental link between name-bearing types and natural populations is correctly established.

## Supplementary Material

XML Treatment for Pristimantis milpe

## Additional information

### Artificial Intelligence (AI) use

The authors accept full responsibility for the content of the manuscript, including the disclosure of any use of AI.

No AI tools were used in the preparation of this manuscript.

### Author ORCIDs

S.R. Ron https://orcid.org/0000-0001-6300-9350

P. Paredes-Aguirre https://orcid.org/0009-0007-8049-9567

D.A. Paucar https://orcid.org/0009-0008-0907-7625

Y. López https://orcid.org/0009-0008-3869-8607

K. Apunte https://orcid.org/0000-0003-4300-5111

J.A. Ortega https://orcid.org/0009-0006-8919-1211

### Data availability

All of the data that support the findings of this study are available in the main text or Supplementary Information.

## Supplementary materials

Supplementary material 1 Specimens examined for this study Data type xlsx Explanation note They are deposited in the herpetological collection of the QCAZ Museum at the Pontificia Universidad Católica del Ecuador, Quito (QCAZ). https://binary.pensoft.net/file/1725124This dataset is made available under the Open Database License (http://opendatacommons.org/licenses/odbl/1.0/). The Open Database License (ODbL) is a license agreement intended to allow users to freely share, modify, and use this Dataset while maintaining this same freedom for others, provided that the original source and author(s) are credited. Santiago R. Ron, Poulette Paredes-Aguirre, Diego A. Paucar, Jhael A. Ortega

Supplementary material 2 Phylogenetic relationships of the *Pristimantis lacrimosus* species group with representative species from other groups of *Pristimantis* Data type pdf Explanation note Maximum likelihood tree obtained for the 16S, 12S, ND1 and RAG1 genes. Branch support (blue numbers) are shown as aLRT values (before the slash) and ultrafast bootstrap values (after the slash). For each individual, the voucher number (or, if unavailable, GenBank accession number) is followed by the species name. https://binary.pensoft.net/file/1725125This dataset is made available under the Open Database License (http://opendatacommons.org/licenses/odbl/1.0/). The Open Database License (ODbL) is a license agreement intended to allow users to freely share, modify, and use this Dataset while maintaining this same freedom for others, provided that the original source and author(s) are credited. Santiago R. Ron, Katherine Apunte

Supplementary material 3 Time-tree of the *Pristimantis lacrimosus* species group with representative species from other groups of *Pristimantis* Data type pdf Explanation note Maximum likelihood time-tree obtained for the 16S, 12S, ND1 and RAG1 genes. Divergence times, in million years (blue numbers) are shown on each node. For each individual, the voucher number (or, if unavailable, GenBank accession number) is followed by the species name. *Pristimantis latidiscus* (= *P. subsigillatus*) is shown in green. https://binary.pensoft.net/file/1725126This dataset is made available under the Open Database License (http://opendatacommons.org/licenses/odbl/1.0/). The Open Database License (ODbL) is a license agreement intended to allow users to freely share, modify, and use this Dataset while maintaining this same freedom for others, provided that the original source and author(s) are credited. Santiago R. Ron, Katherine Apunte
