# Supplementary material for: Long overdue: a new, widespread, species of Pristimantis (Anura, Strabomantidae) from the Chocó region and the synonymy of Pristimantis subsigillatus
Source: Zookeys. 2026 Jul 24;1286:121–52. doi: 10.3897/zookeys.1286.194383 (PMC13428246; doi:10.3897/zookeys.1286.194383)

Supplementary Figure S2. Time-tree of the *Pristimantis lacrimosus species group* with representative species from other groups of *Pristimantis*. Maximum likelihood time-tree obtained for the 16S, 12S, ND1 and RAG1 genes. Divergence times, in million years (blue numbers) are shown on each node. For each individual, the voucher number (or, if unavailable, GenBank accession number) is followed by the species name. *Pristimantis latidiscus* (= *P. subsigillatus*) is shown in green.

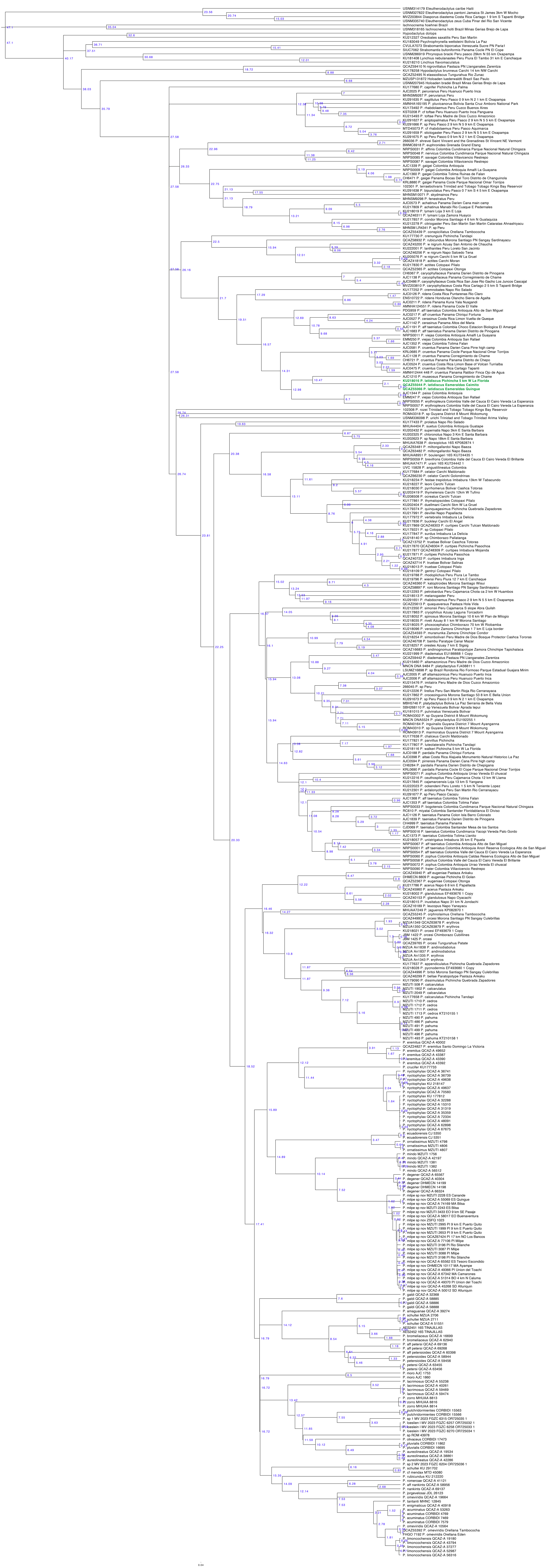

Supplement: Supplementary material 3 — Time-tree of the Pristimantis lacrimosus species group with representative species from other groups of Pristimantis [file zookeys-1286-121_article-194383__-s003.pdf]
