# Supplementary material for: Long overdue: a new, widespread, species of Pristimantis (Anura, Strabomantidae) from the Chocó region and the synonymy of Pristimantis subsigillatus
Source: Zookeys. 2026 Jul 24;1286:121–52. doi: 10.3897/zookeys.1286.194383 (PMC13428246; doi:10.3897/zookeys.1286.194383)

Supplementary Figure S1. Phylogenetic relationships of the *Pristimantis lacrimosus species group* with representative species from other groups of *Pristimantis*. Maximum likelihood tree obtained for the 16S, 12S, ND1 and RAG1 genes. Branch support (blue numbers) are shown as aLRT values (before the slash) and ultrafast bootstrap values (after the slash). For each individual, the voucher number (or, if unavailable, GenBank accession number) is followed by the species name. *Pristimantis latidiscus* (= *P. subsigillatus*) is shown in green.

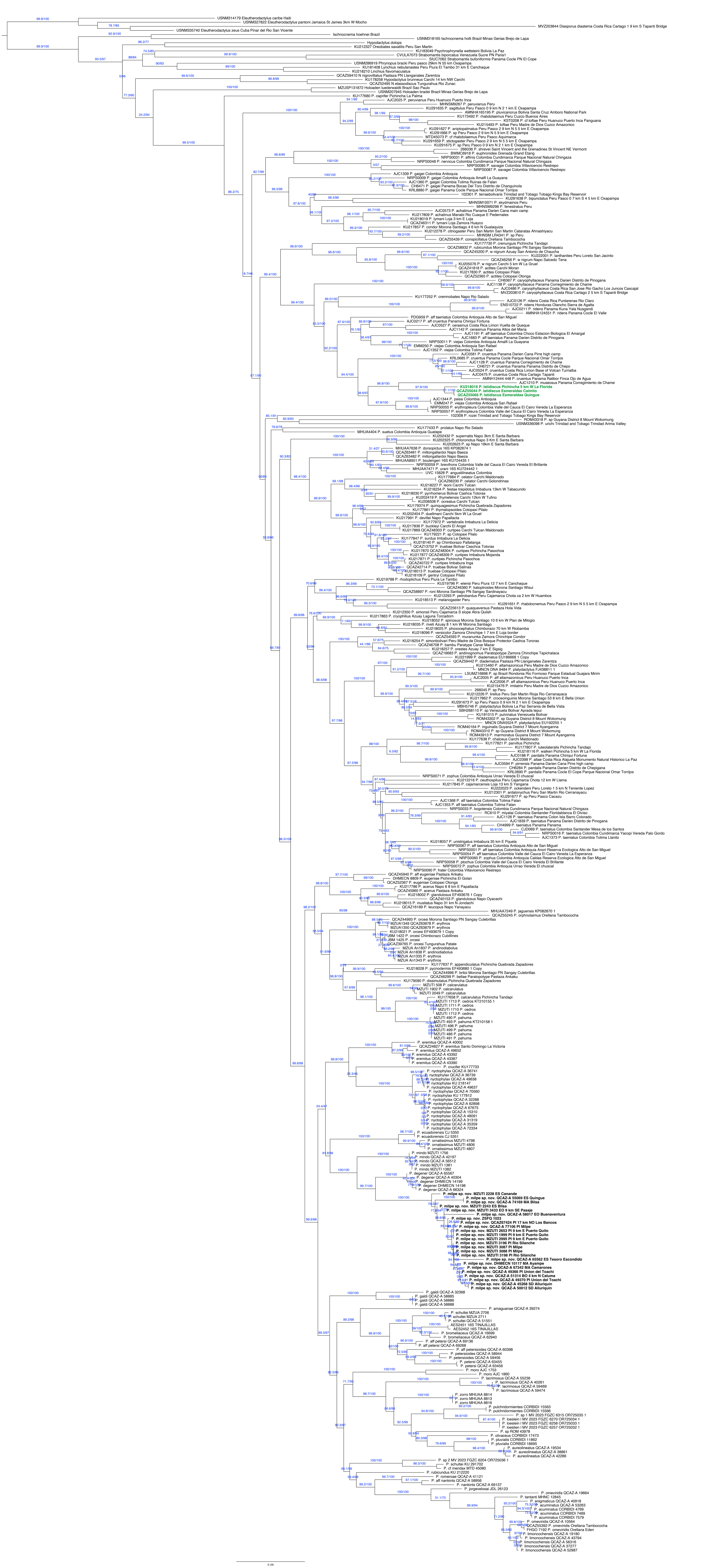

Supplement: Supplementary material 2 — Phylogenetic relationships of the Pristimantis lacrimosus species group with representative species from other groups of Pristimantis [file zookeys-1286-121_article-194383__-s002.pdf]
